# Multiple effects of β-amyloid on single excitatory synaptic connections in the PFC

**DOI:** 10.3389/fncel.2013.00129

**Published:** 2013-09-03

**Authors:** Yun Wang, Thomas H. Zhou, Zhina Zhi, Amey Barakat, Lynn Hlatky, Henry Querfurth

**Affiliations:** ^1^School of Ophthalmology and Optometry and Eye Hospital, Wenzhou Medical UniversityWenzhou, Zhejiang, China; ^2^Steward St. Elizabeth's Medical Center, Tufts Medical School, Tufts UniversityBoston, MA, USA; ^3^Department of Neurology, Rhode Island Hospital, Brown UniversityProvidence, RI, USA

**Keywords:** β-amyloid (Aβ), synaptic connection, synaptic dynamics, excitatory post-synaptic potential (EPSP), short term potentiation (STP), synaptic augmentation (SA), post-tetanic potentiation (PTP)

## Abstract

Prefrontal cortex (PFC) is recognized as an AD-vulnerable region responsible for defects in cognitive functioning. Pyramidal cell (PC) connections are typically facilitating (F) or depressing (D) in PFC. Excitatory post-synaptic potentials (EPSPs) were recorded using patch-clamp from single connections in PFC slices of rats and ferrets in the presence of β-amyloid (Aβ). Synaptic transmission was significantly enhanced or reduced depending on their intrinsic type (facilitating or depressing), Aβ species (Aβ 40 or Aβ 42) and concentration (1–200 nM vs. 0.3–1 μ M). Nanomolar Aβ 40 and Aβ 42 had opposite effects on F-connections, resulting in fewer or increased EPSP failure rates, strengthening or weakening EPSPs and enhancing or inhibiting short-term potentiation [STP: synaptic augmentation (SA) and post-tetanic potentiation (PTP)], respectively. High Aβ 40 concentrations induced inhibition regardless of synaptic type. D-connections were inhibited regardless of Aβ species or concentration. The inhibition induced with bath application was hard to recover by washout, but a complete recovery was obtained with brief local application and prompt washout. Our data suggests that Aβ 40 acts on the prefrontal neuronal network by modulating facilitating and depressing synapses. At higher levels, both Aβ 40 and Aβ 42 inhibit synaptic activity and cause irreversible toxicity once diffusely accumulated in the synaptic environment.

## Introduction

In patients and animal models of the early stages of Alzheimer's Disease (AD), declines in episodic or spatial memory and cognition are correlated with an increase in brain levels of soluble β-amyloid (Aβ) (Lue et al., 1999; Walsh et al., 2002; Rowan et al., 2003). A causal link between the accumulation of Aβ in a soluble, toxic state and impairment of neuronal mechanisms that support memory was demonstrated in a mature β APP transgenic mouse model wherein a single systemic injection of an antibody to Aβ eliminated the memory deficit (Dodart et al., 2002). There is a major focus on the synapse as the initial site of damage in AD (Selkoe, 2002; Nimmrich and Ebert, 2009). Synaptic dysfunction as a consequence of diffusible Aβ is also inferred from studies showing reduced basal transmission and altered plasticity (Klyubin et al., 2005; Shankar et al., 2008; Minano-Molina et al., 2011). In anatomic terms, synapse numbers are reduced early in some AD brain regions (Davies et al., 1987), especially in the prefrontal cortex (PFC) and medial temporal lobe (Morris and Baddeley, 1988). Meanwhile, additional studies in the recent decade indicate that low levels of Aβ peptides could be essential for the modulation of synaptic plasticity (Parihar and Brewer, 2010).

In AD, limbic and association cortices are selectively involved while primary cortical areas remain relatively preserved. These regions of neuronal vulnerability in fact correspond to the degree to which neuronal plasticity can be demonstrated in them (Arendt, 2001). The PFC is a critical association region associated with executive type cognitive function. PFC also orchestrates a unique form of short-term memory termed “working memory.” Working memory is a limited capacity system that supports non-routine types of daily activity. It is a temporary storage system for maintaining and rapidly manipulating information, and is closely connected with attention, strategic information flow and action (Goldman-Rakic, 1996). Experimentally, prefrontal cortical neurons are found to remain persistently active during the delay between sensory cue and an executed response task. The ongoing activity, an electrical correlate of working memory, is stable to the interference from distractors (Goldman-Rakic, 1995). Further, fMRI studies confirm the role of PFC in strategic encoding and goal directed control over the retrieval process in episodic type memory processes (Simons and Spiers, 2003). Less appreciated than episodic memory, working memory is also impaired in the early stages of AD, according to clinical and *in vivo* studies (Morris and Baddeley, 1988). Impairment of PFC function may precede the pathological changes of AD in other cortical association areas (Reid et al., 1996). Correspondingly, soluble Aβ accumulates in the PFC to one of the highest and earliest levels across several cortical regions in the pre-tangle stages of AD (Gouras et al., 2000) and in transgenic mice (Zhuo et al., 2008). Tangle formation in the PFC is also highly correlated with the transition to clearly recognizable dementia (Wang and Al, 2001).

In the PFC network, excitatory synaptic connections in layer V show both facilitated (F-connection) and depressed (D-connection) excitatory post-synaptic potentials (EPSPs) in response to short train stimuli (Wang et al., 2006). F-connections are formed predominantly by complex-type pyramidal cells (PCs) which feature dual apical dendrites, a high degree of interconnectivity and of reciprocity in chemical synaptic connections. In contrast, D-connections are typically formed by simple PCs that are common to primary cortices. A computer simulation study revealed that these facilitating synapses play a crucial role in the formation of persistent neuronal activity, consistent with the properties of working memory (Mongillo et al., 2008). The critical role of the PFC in working memory and early involvement in AD make it a suitable region to examine the electrophysiologic effects of Aβ.

Numerous studies of AD-promoting factors (e.g., Aβ) have examined their effects on field electrophysiological characteristics in the hippocampus and cortex. Because of competing synaptic inputs and influences from other local networks, the use of field recordings may account for seemingly contradictory early reports, where Aβ either increased excitability through membrane depolarization and enhancing long term potentiation (LTP) or depressed both synaptic transmission and LTP induction (Wu et al., 1995; Selkoe, 2002; Walsh et al., 2002; Esteban, 2004; Puzzo et al., 2008; Li et al., 2011). Up until now, a study focused on activity-dependent plasticity specific to the association cortex has not been reported. It is therefore timely to carry out an investigation of an *in situ* neural network, especially at the resolution of individual synaptic connections, within an association cortical area such as the PFC.

In this study, using multi-neuron patch clamp recording from PFC slices, we found that synaptic responses of single excitatory synaptic connections were significantly enhanced or reduced depending on their intrinsic type (facilitating or depressing), the tested Aβ species (40 or 42 amino acids) and concentration (low dose 1–200 nM vs. high dose 0.3–1 μ M). Low-doses of Aβ 40 enhanced F-connections and inhibited D-connections in the PFC. In contrast, high-doses of Aβ 40 and low-doses of Aβ 42 inhibited all types of excitatory synaptic connections. Further, the inhibition induced with bath application was commonly difficult to recover or even became worse by washout. However, direct local and brief application of the peptides by pipette at comparable concentrations produced similar inhibitions with a rapid and complete recovery upon washout. Based on the principles of synaptic dynamics that have been well-studied in our previous computer simulation of synaptic responses of single synaptic connections, the effects of Aβ were considered to be produced via both pre- and post-synaptic mechanisms.

## Materials and methods

### Electrophysiological recordings

Prefrontal cortical slices were prepared using a published protocol (Wang et al., 2006). Briefly, brain was dissected from normal adolescent Wistar rats (day 25–35) or young adult ferrets (7–9 weeks old). PFC slices (300 μ M) were sectioned using a vibratome (DTK 1000 Zero 1 Microslicer) and then incubated in artificial cerebrospinal fluid (ACSF) before transfer to a recording chamber (at 34°C). Neurons in layer V of the medial PFC were visually identified using infrared differential contrast videomicroscopy (BX50WI, Olympus). An advanced technique consisting of quadruple patch clamp recording was used to record from candidate cell bodies (somata), and single synaptic connections formed between neuron pairs were determined electrophysiologically according to standard characteristics of chemical synaptic transmission (Wang et al., 2006). Somatic whole-cell signals (6–12 mΩ pipette resistance) were amplified using Axoclamp-200B amplifiers (Axon Instruments, USA). Recordings were sampled over real time and filtered using the program Igor (Igor Wavemetrics, Lake Oswego, OR, USA), digitized by an ITC-18 interface (Instrutech, Great Neck, NY, USA) and stored on hard drive (Macintosh G5 computer) for off-line analysis (Igor). Stimulating and voltage recording glass micropipettes were filled with (mM): 100 potassium gluconate, 20 KCl, 4 ATP-Mg, 10 phosphocreatine, 0.3 GTP, 10 Hepes (pH 7.3) and 0.4% biocytin (Sigma). Presynaptic action potentials (AP) were elicited using short (3 ms), suprathreshold, intracellular depolarizing current pulses. The extracellular recording solution consisted of ACSF, containing (mM): 125 NaCl, 2.5 KCl, 25 glucose, 25 NaHCO_3_, 1.25 NaH_2_PO_4_, 2 CaCl_2_, and 1 MgCl_2_. Only neurons with stable access resistance were included in the statistical analyses. Membrane potentials were routinely voltage-clamped at −70 ± 2 mV to maintain Vm against drift by using small current injections. Neurons were filled with 0.4% biocytin (Sigma) by diffusion at the end of the recordings for later identification of neuronal types.

Once synaptic connections were obtained, the EPSP failure rate, certain dynamic features of EPSPs and short term potentiation components including synaptic augmentation (SA) and post-tetanic potentiation (PTP), were recorded (Wang et al., 2006). For the EPSP failure rate, single APs were repeatedly evoked (0.5 Hz, 15–30 times) in a presynaptic cell and the number of corresponding EPSP failures in a postsynaptic cell were counted. For the synaptic dynamic features, an EPSP train was evoked by 6–8 presynaptic APs at 10–20 Hz followed by a recovery test response (RTR) after a 500 ms delay. SA and PTP were induced by giving a 15 pulse (tetanus) stimulus at 50 Hz. Single test responses (0.5 Hz) were recorded for 20 s before and up to 100 s after the train. This procedure was repeated four times, each preceded by a 2 min interval.

### Histological procedures and 3D computer reconstruction

After recording, the slices bearing biocytin-injected neurons were fixed for at least 24 h in cold 0.1 M phosphate buffer saline (PBS, pH 7.4) containing 2% paraformaldehyde, 1% glutaraldehyde, and 0.3% saturated picric acid. Thereafter, the slices were rinsed several times (10 min each) in PBS. To block endogenous peroxidases, slices were transferred into phosphate-buffered 3% H_2_O_2_ for10–30 min. After five to six rinses in PBS (10 min each), slices were incubated overnight at 4°C in avidin-biotinylated horseradish peroxidase according to the manufacturer's protocol (ABC-Elite, Vector Labs, Petersborough, UK) (2% A, 2% B, and 1% Triton-100). Following incubation and additional rinses, the reaction was developed with diaminobenzidine (DAB) under visual control using a bright-field microscope (Zeiss, NY, USA) until all cell processes appeared clearly visible (usually after 2–4 min). The reaction was stopped upon transferring the sections into PBS. Slices were mounted in aqueous mounting medium.

3D neuron models were reconstructed from stained cells using the Neurolucida system (MicroBrightField Inc., USA) and a bright-field light microscope (Olympus, BX51, Japan). Reconstructed neurons subsequently underwent quantitative analysis using the NeuroExplorer (MicroBrightField Inc., USA; ×60 magnification, numerical aperture = 0.9; Z-axis resolution = 0.37 μm). Putative synapses were identified according to the criteria as published (Wang et al., 2002).

### Preparation and treatment of soluble synthetic Aβ

**Figure A1:**
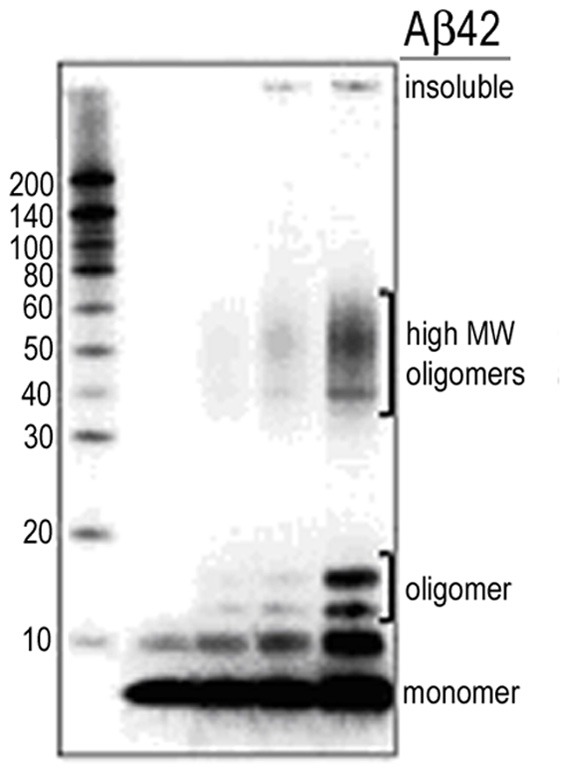
**Western blot monitoring of Aβ stock solutions**. Monomer predominant preps as shown in lane 1 were used for the recording of single synaptic connections. Synthetic Aβ peptides were solubilized in 1,1,1,3,3,3-hexafluoro-2-propanol (HFIP), aliquoted, dried and stored at −80°C. A working stock solution (0.1 mM) was then prepared and stored at −80°C for dilution immediately before use. Aged or oligomerized preps as appear in lane 5 were not used in the current work.

Soluble synthetic Aβ peptides (Aβ 40, Aβ 25–35, and Aβ 42) were purchased from Biosource (Camarillo, CA) or the Harvard Protein Core laboratory and prepared as 0.1 mM stock following published methods and stocked at −80°C (Stine et al., 2003). The quality of monomer stock solutions were examined periodically using western blots [NuPAGE Novex 4–12% Bis-Tris gels (Invitrogen)] (Figure A1). Mouse monoclonal anti-human amyloid beta protein antibody (6E10, purchased from Signet) was used at a 1000X dilution. Horse anti-mouse IgG HRP-linked antibody (Cell Signaling Technology, 2000X dilution) was used as secondary antibody and the development with Chemiluminol that followed was captured on hyperfilm (Amersham Biosciences). Different concentrations of Aβ were freshly prepared before use by defrosting and diluting the stock solution with ACSF. Only monomer predominant stock solution was used in the current study. Soluble Aβ was bath-applied either at low dosage (1–200 nM) or at high dosage (300 nM–1 μ M) (see Chen et al., 2000). During recording, brain slices were continuously perfused with oxygenated ACSF at a flow rate of 0.75–1.0 ml/min. The ACSF volume in the tube leading to and including the recording chamber was 1.5–2.0 ml. This enabled quick replacement of the recording solutions (≤3 min.) when switching between experimental procedures. Each recording procedure was repeated under three conditions: (1) pre-application, (2) application, and (3) washout of Aβ. The Aβ application recordings typically lasted for 30 min. whereas washout recordings lasted for 10–30 min.

### Modeling analysis of synaptic responses

The quantitative analysis of basal synaptic dynamic properties of excitatory connections has been carried out using a well-known computer model of a combination of EPSP train and a RTR evoked with a 500 ms delay (Markram et al., 1998; Tsodyks et al., 2000; Wang et al., 2006). The RTR is used to test the recovery of synaptic facilitation or depression, which characterizes the synapse type. The model extracts four key parameters of the connection: *DFUA* (*D*, the time constant of recovery from depression (ms); *F*, the time constant of recovery from facilitation (ms); *U*, utilization of synaptic resources, analogous to the neurotransmitter release probability (*p*); *A*, the absolute strength of a synaptic connection (nA), defined as its maximum response when *p* = 1). This modeling approach is based on fitting the mean output behavior of synaptic connections and therefore requires inputting only averaged responses (i.e., average EPSP traces). Generally speaking, reductions in *A* correspond to the situation when the amplitudes of all EPSPs in the train and RTR become smaller, keeping an unchanging EPSP pattern. In the case of reductions in *U*, the amplitude of the 1st EPSP is reduced whereas subsequent EPSPs are facilitated. When normalized to the first EPSP in such recordings, the subsequent EPSP amplitudes, but not the RTR, are magnified. In the case of a larger *D* value, both EPSPs (subsequent to the 1st) and the RTR show reductions. Oppositely, a high value for the parameter *F* correlates to the situation in which both EPSPs (subsequent to the 1st) and the RTR are increased.

### Statistical analysis

Paired student *t*-test was used to compare EPSP responses between different conditions: [(1) pre-application, (2) application, and (3) washout of Aβ.]. Unpaired student *t*-test was used for the comparison of EPSP responses between different treatments of Aβ. The statistical analyses of EPSP train, SA and PTP were all based on an intrinsic comparison of individual single synaptic connections between the Aβ application or washout condition with the pre-application condition. In order to lessen the influence from the variance of synaptic strength between individual connections, the statistical comparisons were made using normalized values. In the analysis of EPSP train, all EPSP values of a connection were normalized to the mean of EPSPs recorded under the pre-application condition. In the analysis of SA and PTP, all EPSP values of a connection were normalized to the mean of pre-tetanus EPSPs obtained in the pre-Aβ application condition. Furthermore, an EPSP pattern defined by a certain “8-EPSP train + RTR” configuration for each type of synapse, allowed each EPSP value to be treated as an independent outcome value in the statistical comparison. Multiple outcome values per connection were therefore used in the EPSP train analysis. The same principle was also applied to the analyses of SA and PTP.

## Results

**Figure 1:**
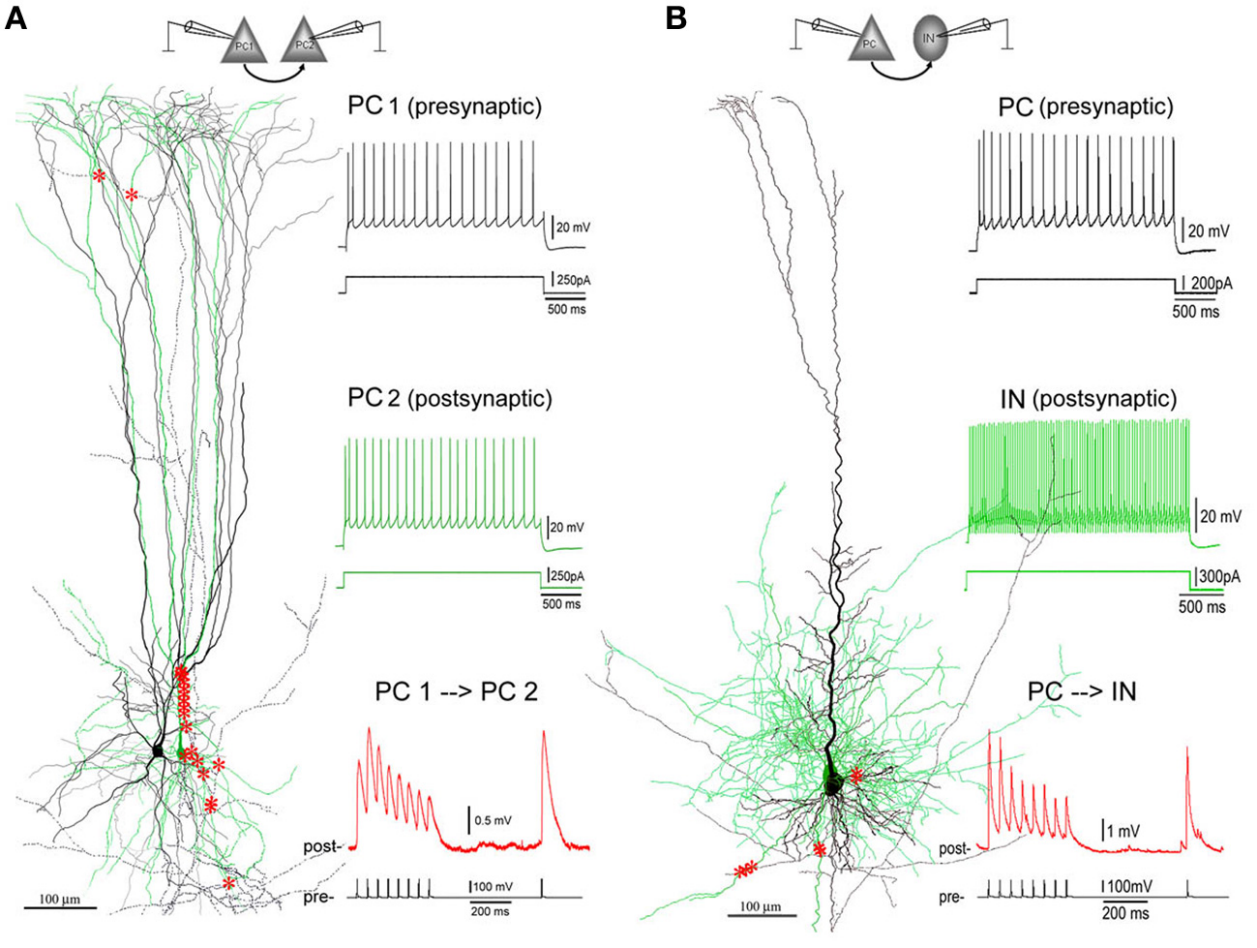
**Excitatory synaptic connections in layer V of PFC. (A)** A facilitating (F-type) PC-PC connection in layer V of the PFC of an 8-weeks old ferret. Left panel: 3D computer reconstruction of the connection: Both pre- (PC1 in black) and post-synaptic (PC2 in green) cells are complex PCs featured by an apical dendrite with multiple early-bifurcated major branches. A total of 20 putative synapses are marked with red stars onto the basal, apical, oblique and tuft dendrites of PC2. Right panel: Physiological responses of pre- (upper, in black) and post-synaptic (middle, in green) cells were induced by injections of depolarization currents into their somata. Excitatory postsynaptic potentials (EPSPs, down, in red) were recorded from PC2 by giving brief current injections to induced action potentials (APs, bottom, in black) in PC1. The postsynaptic response train is composed of 8 EPSPs at a 20 Hz frequency followed by a recover test response (RTR) with 500 ms delay. **(B)** A depressing (D-type) PC-IN connection in layer V of the PFC of a P30 rat. The color coding for the reconstructed pre- and post-synaptic cells and their physiological and synaptic responses are the same as the PC-PC connection in **(A)**. A total of 7 putative synapses are marked with red stars onto basal dendrites of the postsynaptic interneuron. Note: The postsynaptic interneuron appears to be a fast-spiking basket cell according to its axonal and dendritic morphologies and fast AP firing induced by depolarization current injection to its soma.

Quadruple patch clamp recording was performed to record synaptically coupled pairs (*n* = 100) in layer V of the PFC of rats (*n* = 92, age P25–P35) and ferrets (*n* = 8, age 7–9 weeks old). Since synaptic dynamics (i.e., depressing and facilitating types) of synapses are consistent across species (Wang et al., 2006), the data of the two species were pooled together in order to maximally utilize the obtained data. The studied connections comprise synapses formed between PCs (PC–PC, *n* = 86 pairs) and those formed by a PC onto an interneuron (PC–IN, *n* = 14 pairs) (Figure 1). The neuronal type was identified according to the morphology (PCs and interneurons—mainly basket cells and Marttinoti cells) combined with the firing pattern of APs evoked by depolarizing current steps injected into neuronal somata (Wang et al., 2002, 2006). EPSP responses of a postsynaptic cell were induced by APs evoked by brief depolarizing current injections delivered into the presynaptic neuronal soma. We previously characterized the excitatory neuronal network in layer V of the PFC (Wang et al., 2006). Facilitation-dominant synapses (F-connections) are abundant in the PFC while depression-dominant synapses (D-connections), typically common in primary cortical areas, form a minor population in the PFC. In order to examine effects of beta-amyloid peptides (Aβ 40, Aβ 25–35, Aβ42) on single F- and D-connections, low- (1–200 nM) and high-dosages (0.3–1 μ M) of Aβ were continuously bath-applied while recording of EPSPs. The failure rate, synaptic dynamics and STP were investigated. The experimental procedure of applying Aβ was successfully performed in 48% of recorded connections (48 out of 100 pairs: rats, PC–PC, *n* = 35 pairs and PC–IN, *n* = 7 pairs; ferrets, PC–PC, *n* = 6 pairs). Unfortunately, synaptic responses of the other 52 connections became unstable or even disappeared in the middle of Aβ application. These unstable connections were excluded from the data analysis.

### Influences of Aβ on failure rates of F- and D-connections

**Figure 2:**
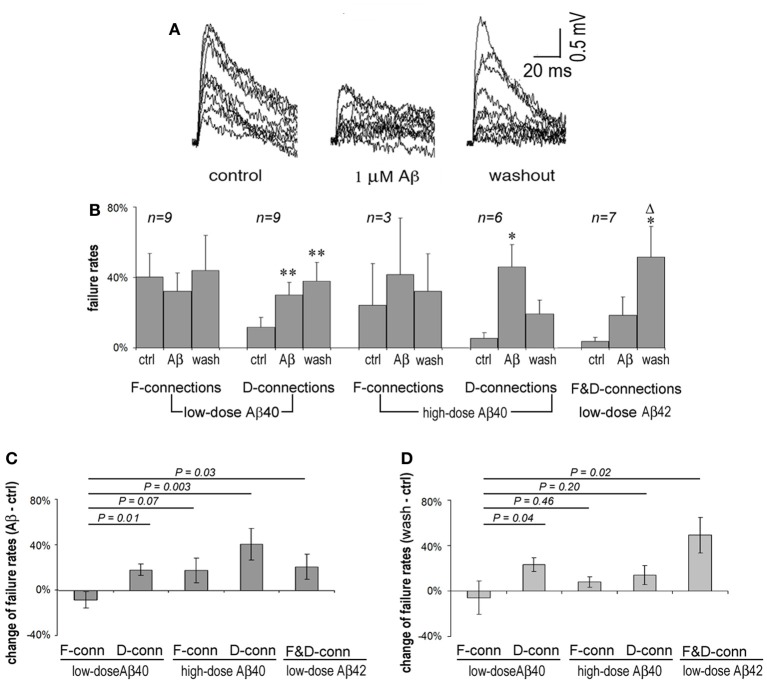
**Synaptic failure rates vary according to synaptic type and upon Aβ species and concentration. (A)** Superimposed 15 single EPSP traces recorded at 0.5 Hz from an F-type connection in pre-application, application and washout phases of 1 μ M Aβ 40. EPSPs were generally reduced and the number of failures increased during application of Aβ for 20 min, which tended to recover on washout for 10 min. **(B)** Average failure rates of F- and D-connections in pre-application, application and washout phases in the presence of Aβ were charted according to Aβ species and concentration, and connection type. The synaptic failure rate tended to decrease in F-connections after low-dose Aβ 40 applied. In contrast, the failure rates appear to increase in all other cases. Low and high doses of Aβ 40 applied to D-connections reached significance (*P* = 0.01 and *P* = 0.024, respectively). A trend to enhance failure rates is shown in the case of high-dose Aβ 40 to F-connection (*n* = 4) and low-dose Aβ 42 to F- (*n* = 3) and D-(*n* = 4) connections. After washout for 10–30 min., the failure rate are further exacerbated in applications of low-dose Aβ 42 to F- and D-connections (compared with pre-application, *P* = 0.04; compared with Aβ application, *P* = 0.05). Note: ^*^compared with pre-application, ^Δ^compared with Aβ application; ^*^ or ^Δ^ *P* < 0.05; ^**^ *P* < 0.01. **(C)** Net changes in average failure rates following exposure to Aβ (the failure rate in Aβ application - the failure rate in pre-application). The net rate change in low-dose Aβ 40 to F-connections was opposite in direction to that of the other cases. The difference between the net rate changes corresponding to low-dose Aβ 40 vs. high-dose Aβ 40 to F-connections did not quite reach statistically significance possibly due to the low *n* (*n* = 3) in the latter case. **(D)** Net changes to average failure rates by washout of Aβ (the failure rate in washout of Aβ-the failure rate in pre-application). The differential change in failure rates remained virtually similar to that in **(C)**. Notably, the net rate change became smaller (from 41 to 14%) in high-dose Aβ 40 to F-connection, but became bigger (from 21 to 49%) in the cased of low-dose Aβ 42 to F-and D-connections.

Presynaptic APs can fail to induce the release of neurotransmitter resulting in failures of evoked EPSPs. F-connections generally display higher failure rates than do D-type connections because the initial release probability of F-connections is usually lower. Single APs in presynaptic cells were generated at 0.5 Hz and EPSP failure rates of F- and D-connections were observed, respectively, under conditions of pre-application (in ACSF only), Aβ application and washout. An example of widening transmission failure in an F-type synapse exposed to 1 μ M Aβ 40 and moderate recovery upon washout is given in Figure 2A. Surprisingly, we found that lower doses of Aβ 40 tended to reduce the synaptic failure rate in F-connections. In contrast, the transmission failure rate became increased under all other studied conditions, including low-dose Aβ 40-bathed D-connections, high-dose Aβ 40 or low-dose Aβ 42 applied to F- or D-connections (Figure 2B). The increase in failure rate was statistically significant for the cases of low and high doses of Aβ 40 to D-connections (*P* = 0.01 and *P* = 0.024, respectively). A trend toward higher failure rate, although not statistically significant, was clearly visible in the cases of high-dose Aβ 40 to F-connection (*n* = 3) and low-dose Aβ 42 to F- (*n* = 3) and D- (*n* = 4) connections. The opposing directions in failure of synaptic responses was present in Figure 2C, wherein, the net failure reduction in the case of low-dose Aβ 40 to F-connections was opposite in direction to the net failure increase in all other cases. After washout for 10–30 min, these contrasting net changes in failure rates virtually remained (Figure 2D). Note the failure rate was further increased in the case of low-dose Aβ 42 to F- and D-connections following washout (Figure 2B, far right set: compared with pre-application, *P* = 0.04; compared with Aβ application, *P* = 0.05). This phenomenon indicates that Aβ 42 is selectively more toxic to synaptic connections in the PFC.

### Differential effects of Aβ on synaptic dynamics of F- and D-connections

**Figure 3:**
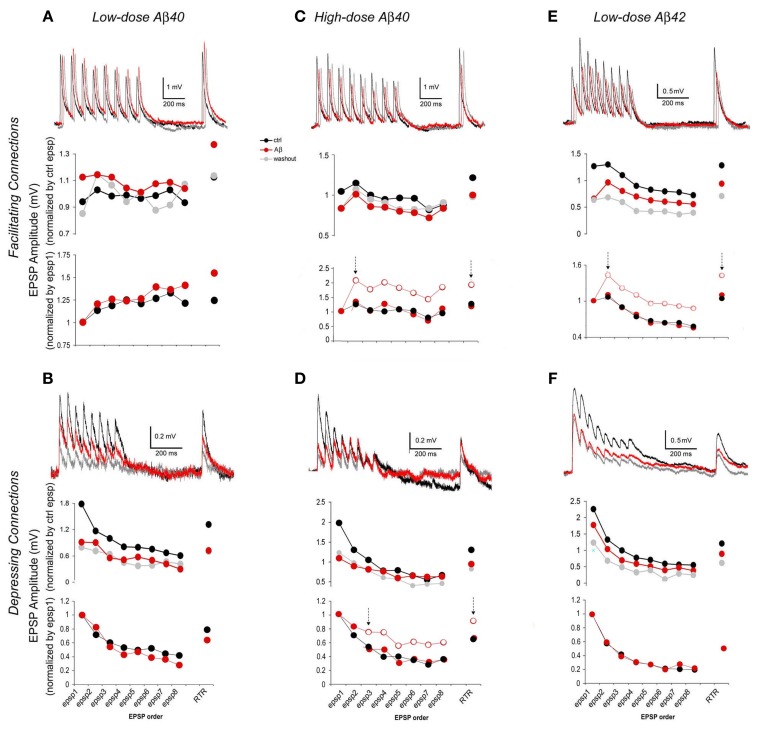
**EPSP trains of F- and D- connections change differentially depending upon the synapse type and the Aβ species and concentration**. In each case, representative traces (each was an average of 15–30 individual traces) from pre-application, Aβ application and washout phases are presented at the top of each graph. The chart in the middle gives average EPSP amplitudes that were normalized to the mean of pre-application EPSPs for the comparison between pre-application and Aβ application and washout. The chart at the bottom alternatively shows average EPSP amplitudes that were normalized instead to the 1st EPSP of their intrinsic run in order to access changes in EPSP patterns (for clarity, traces of washout were not plotted). **(A)** Low-dose Aβ 40 enhanced F-connections. The overall increase in the EPSP train was followed by a comparably larger increment in the RTR. The enhancement tended toward recovery after washout. **(B)** Low-dose Aβ 40 inhibited D-connections. The EPSP amplitudes were all significantly diminished while the EPSP pattern remained virtually similar to that in pre-application. **(C)** High-dose Aβ 40 inhibited F-connections. EPSPs were unevenly reduced, in which the decrement of the 1st EPSP was greater. In the chart at the bottom, the empty circles between two arrows showed the real pattern of subsequent EPSPs as they relate to the 1st EPSP. The red dots between two arrows that were disassociated with the 1st EPSPs, show a match up of patterns of subsequent EPSPs and RTR between pre-application and Aβ application conditions. **(D)** High-dose Aβ 40 inhibited D-connections. The 1st EPSP was notably reduced while the amplitudes of steady state EPSPs (4th through 8th EPSPs) remained unchanged, followed by a reduced RTR. Between two arrows in the chart at the bottom, the empty circles show the pattern of 3rd through 8th EPSPs + RTR as they relate to the 1st and 2nd EPSPs, and the red dots between two arrows were disassociated with the 1st and 2nd EPSPs, giving the matching patterns of 3rd–8th EPSPs and RTR between pre-application and Aβ applications. **(E)** Low-dose Aβ 42 inhibited F-connections. EPSPs were unevenly reduced, also indicating that the decrement of the 1st EPSP was greater (The same chart presentation was made as in **C**). **(F)** Low-dose Aβ 42 inhibited D-connections. EPSPs were all significantly diminished while the EPSP pattern remained almost the same as in pre-application. Note: Upon washout of Aβ (see the middle charts, also see Table 1), the enhancement of low-dose Aβ 40 to F-connections (in **A**) appeared to recover to the pre-application level while EPSPs inhibited by Aβ did not recover in **B–D** or even further diminished in **E** and **F**.

**Figure A2:**
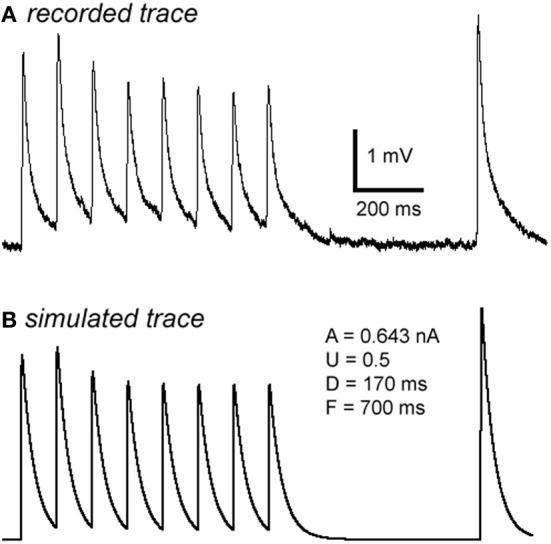
**Computer simulation of synaptic responses**. The 10 Hz EPSP train followed by a RTR with 500 ms delay was recorded from a PC-IN connection **(A)**. Using a model of dynamic synaptic transmission, the synaptic dynamics were assessed by fitting the EPSPs **(B)**. The modeling extracts four key parameters of the connection: *DFUA* (*D*, the time constant of recovery from depression; *F*, the time constant of recovery from facilitation; *U*, utilization of synaptic resources used analogously to release probability, *p*; *A*, the absolute synaptic strength). If samplings were big enough, these parameters could be quantitatively analyzed enabling a quantitative comparison of basal synaptic dynamic changes induced by Aβ. Trends of changes induced by Aβ were revealed despite low samplings in most data sets (see Table A1).

EPSP trains generated by 5–8 APs and RTR 500 ms later were used in a phenomenological modeling strategy to estimate dynamic synaptic parameters—*AUDF* (Markram et al., 1998; Tsodyks and Markram, 1997; Wang et al., 2006). Dynamic synaptic responses are due to the interplay between *U*, *D*, and *F*. *U* represents the probability of synaptic transmitter release, *p*. *D* is the time constant to recover from synaptic depression; *F* is the time constant to recover from synaptic facilitation. The absolute strength, *A*, of a synaptic connection is defined as the maximum synaptic response when *p* equals 1. This approach is based on the mean output behavior of synaptic connections and therefore requires analyzing only average responses (Figure 3 upper row graphs). Fitting average responses of an EPSP train into the model yields values for *AUDF* parameters of a synaptic connection (Figure A2 and Table A1) (Tsodyks and Markram, 1997). According to the principle as verified in our previous studies (Tsodyks and Markram, 1997; Markram et al., 1998; Wang et al., 2006), changes in the synaptic parameters *AUDF* are essentially evaluated based on the amplitudes and the amplitude pattern of average EPSP train and RTR. Generally speaking, in the case of *A* reduction, amplitudes of all EPSPs become smaller but the EPSP pattern remain unchanged. In the case of *U* reduction, the amplitude of 1st EPSP is reduced while subsequent EPSPs, but not RTR, are facilitated. In the case of *D* enhancement, both subsequent EPSPs and RTR are reduced. In the case of *F* enhancement, both subsequent EPSPs and RTR are increased.

In accordance to this model (Tsodyks and Markram, 1997; Markram et al., 1998; Wang et al., 2006), EPSP trains evoked by 5–8 presynaptic APs and a RTR recorded after a 500 ms delay were analyzed for the estimation of synaptic dynamics based on their amplitudes and patterns of EPSPs, respectively, in pre-application, various Aβ application and washout conditions (Figure 3). For comparison between pre-application and Aβ application and washout, average EPSP amplitudes of individual synaptic connections were first normalized to the mean of EPSPs in pre-application conditions (Figure 3 middle row graphs) of either low dose Aβ 40 (Figures 3A,B) or high dose Aβ 40 (Figures 3C,D) or low dose Aβ 42 (Figures 3E,F) applications, respectively. Next, in order to better present changes in EPSP patterns, the same responses were alternatively normalized to the 1st EPSP of their own trains (Figure 3 lower row graphs).

Compared with the pre-application, EPSP amplitudes were significantly increased in F-connections exposed to low-dose Aβ 40 (Figure 3A, *n* = 12 pairs). The amplitude increase of the EPSP train was followed by a comparably larger increment in the RTR (Figure 3A lower row graph), which indicated the enhanced facilitation, *F*. In all other conditions examined (low-dose Aβ 40 to D-connections, high-dose Aβ 40 to F- or D-connections and low-dose Aβ 42 to F- or D-connections) (Figures 3B–F), the EPSP amplitudes were all significantly diminished compared to their own pre-applications, respectively, (Table 1). In both low-dose Aβ 40 and low-dose Aβ 42 to D-connections (Figure 3B, *n* = 7 pairs; Figure 3F, *n* = 5 pairs), the amplitudes of EPSPs were evenly reduced and the EPSP pattern virtually remained the same as in the pre-application. This change is represented as a typical reduction in the absolute synaptic strength, *A*. Interestingly, high-dose Aβ 40 and low-dose Aβ 42 in F-type connections (Figure 3C, *n* = 4 pairs; Figure 3E, *n* = 8 pairs) similarly resulted in an uneven reduction of EPSP trains and RTR, in which the decrements of the 1st EPSPs were greater. This result indicated a reduction in absolute synaptic strength, *A* (according to a decline in all EPSPs) accompanied by a reduced release probability, *U* (according to the greater decrements of the 1st EPSPs). In the high-dose Aβ 40 to D-connections (Figure 3D, *n* = 7 pairs), the 1st EPSP was notably reduced while the amplitudes of steady state EPSPs (4th–8th EPSPs) remained unchanged. The notable decrement of the 1st EPSP represented a *U* reduction, which typically leads to an immediate facilitation of subsequent EPSPs of the train. However, such an immediate facilitation was not visible. Instead, the unchanged steady state EPSPs was followed by a reduced RTR. This phenomenon could be attributed to an interplay of the reduction in both *U* and *A*. The immediate facilitation of subsequent EPSPs due to the notable *U* reduction, would counterbalance the reduction of these EPSPs due to the reduction of parameter *A*, keeping them unchanged.

**Table 1:** **Comparison results of EPSP trains recorded in pre-application, application and washout of Aβ**.

	**low-dose Aβ40**		**high-dose Aβ40 and Aβ 25–35**		**low-dose Aβ42**	
	**F-connection**	**D-connection**	**F-connection**	**D-connection**	**F-connection**	**D-connection**
ctrl vs. Aβ	*P* = 0.02	*P* < 0.001	*P* < 0.001	*P* < 0.001	*P* < 0.001	*P* < 0.001
ctrl vs. washout	*P* = 0.96	*P* < 0.001	*P* < 0.001	*P* < 0.001	*P* < 0.001	*P* < 0.001

*Paired student t-test was used with multiple outcome values per connection*.

High-dose Aβ25–35 was used in a few test recordings considering the fact that this short peptide has neurotoxic action and aggregating property (Chen et al., 2000). The high-dose Aβ 25–35 showed inhibiting effects on EPSP trains of F- (*n* = 2 pairs) and D-connections (*n* = 3 pairs). The changes in EPSP train induced by Aβ 25–35 were similar to those induced by the high-dose Aβ 40.

### Effects of Aβ are fully reversible when applied briefly and locally

**Figure 4:**
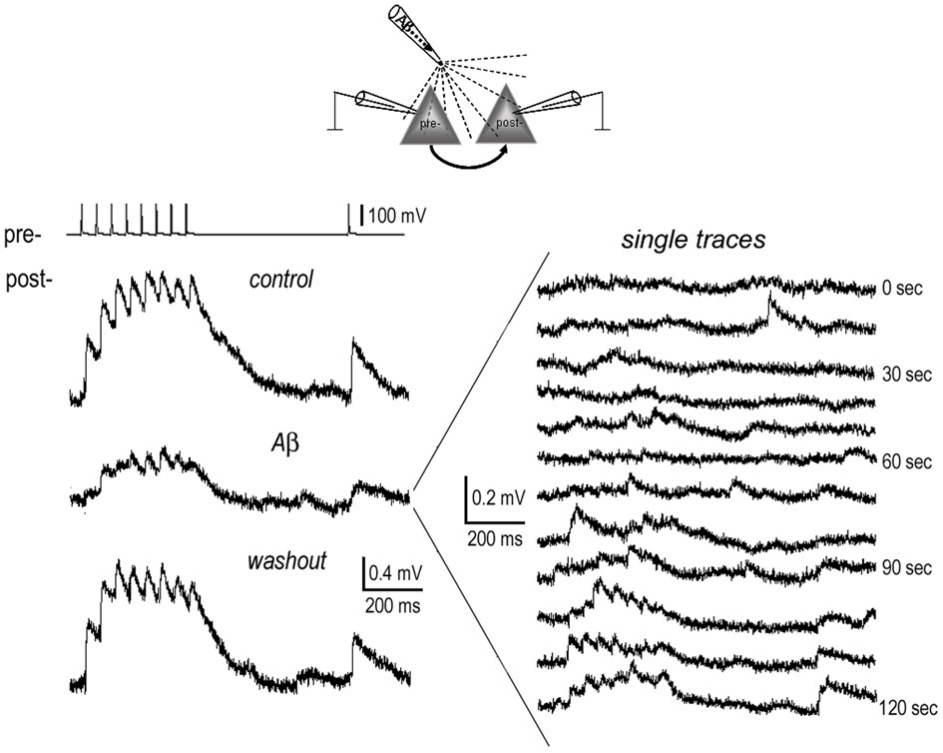
**Full recovery of EPSPs from inhibition by brief local application of Aβ**. 1 nM Aβ 42 was briefly applied near the somata of the connected neurons (diagram) for 2 min. Recordings were carried out before (pre-application) and at the end of application of Aβ, and lastly after washout of Aβ for 10 min. The average EPSP traces display full recovery from the inhibition of Aβ (**left panel**). In the single traces recorded at the end of Aβ application (**right panel**), the EPSPs almost completely disappeared. These started to recover at ~1 min. after terminating the Aβ “puff.”

It is noteworthy that upon washout of the various Aβ-containing mediums, only the enhancement of F-connections by low-dose Aβ 40 recovered (*P* > 0.05 in Table 1, Figure 3). The reductions in EPSP train and RTR in all the other cases did not (*P* < 0.05 in Table 1). Rather than reflecting inefficient washout, we suspect a damaging effect on synapses was induced by prolonged Aβ conditions such as in the presence of high concentrations of Aβ 40 or low-dose Aβ 42. To test this, we briefly applied 1nM Aβ 42 (*n* = 2) or 1 μ M Aβ 40 (*n* = 1) locally to a synaptic connection via a “puff” using a 3rd pipette (a representative recording is shown in Figure 4, estimating that the local concentration of Aβ remained close to the concentration in the pipette). Recordings were obtained prior to and at the end of peptide application (only 2 min) and bath washout phases (10 min). The synaptic responses almost completely disappeared at the end of application of Aβ. The recovery of EPSPs, however, began quickly at ~1 min after the application phase of Aβ had terminated and was largely recovering from inhibition within 2 min into the washout (Figure 4, right panel). A full recovery was observed 10 min into the washout (Figure 4, the bottom trace in left panel). These results suggest that brief, highly local exposures of synapses to Aβ (even at a high level of concentration) produce reversible inhibition. With this evidence, the aforementioned irreversible inhibitory effects of bath-applied peptides becomes understandable if either modest diffuse accumulations of Aβ 42 or abnormally high levels of Aβ40, may be enough to damage synapse function.

### Opposite effects of low nanomolar Aβ40 and Aβ42 on short term potentiation of F-connections

**Figure 5:**
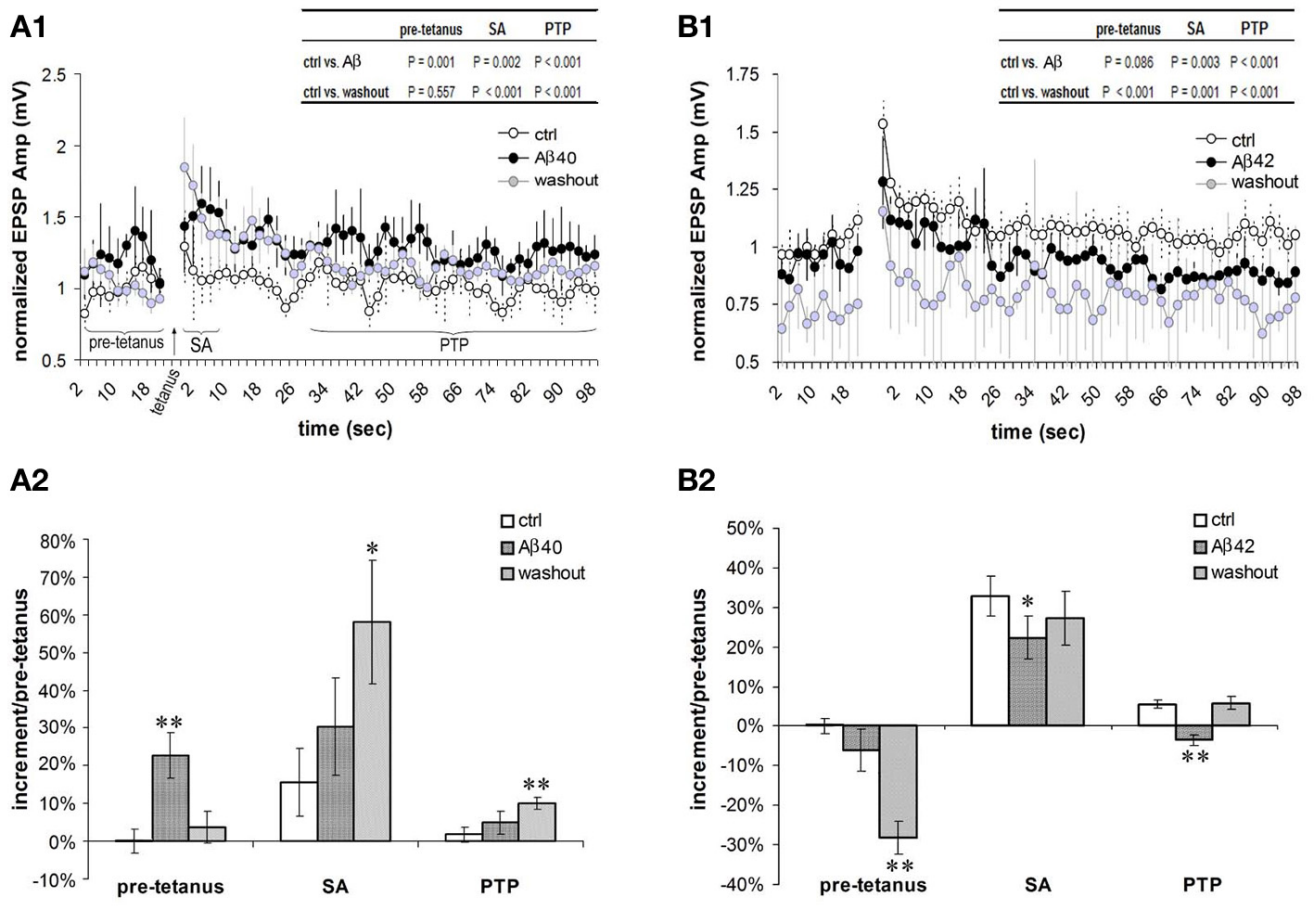
**Differential effects of low nanomolar Aβ 40 and Aβ 42 on the SA and PTP. (A1)** Low-dose Aβ 40 enhanced synaptic responses (i.e., EPSPs) under all the recording phases (pre-tetanus baseline, SA induction and PTP induction) in F-connections (all *P* < 0.01, *n* = 4 pairs). After washout for 10–30 min, the EPSPs during the pre-tetanus phase recovered to the pre-application level (*P* = 0.557), but still remained significantly higher during the SA and PTP induction phases (both *P* < 0.01, inset table). EPSP amplitudes were normalized to the mean of pre-tetanus EPSPs in pre-application. Paired *t*-test with multiple outcome values per connection was performed between pre-application and Aβ application, and between pre-application and washout phases. **(A2)** Comparison of increments during pre-tetanus, SA and PTP induction in the case of low-dose Aβ 40 application. Compared with the baseline level (0 ± 3%) of increment during pre-tetanus phase of pre-application condition, low-dose Aβ 40 enhanced the average baseline EPSP by 23 ± 6% (*P* = 0.01), recovering after a 10–30 min washout (4 ± 4%, *P* = 0.556). Compared with 16 ± 9% in pre-application, the SA appeared to be enhanced by low-dose Aβ40 to 30% ± 13% (*P* = 0.141), and remained enhanced at an average level of 58 ± 16% after a 10–30 min. washout (*P* = 0.05). Similarly, compared with 2 ± 2% in pre-application condition, the PTP appeared to be enhanced by low-dose Aβ40 to 5 ± 3% (*P* = 0.284), and remained enhanced to a statistically significant level after a 10–30 min. washout (10 ± 2%, *P* = 0.01). **(B1)** Low-dose Aβ 42 depressed synaptic responses at the pre-tetanus baseline, and significantly at the SA and PTP inductions (both *P* < 0.01, *n* = 6 pairs). After a 10–30 min. washout, the EPSPs under all the recording phases (pre-tetanus, SA induction and PTP induction) became significantly depressed (*P* < 0.01, inset table). **(B2)** Comparison of increments during pre-tetanus, SA and PTP inductions in low-dose Aβ 42 applications. Compared with the baseline level (0 ± 2%) of increment during the pre-tetanus phase of pre-application condition, low-dose Aβ 42 depressed the average baseline EPSP by −6 ± 5% (*P* = 0.08). This became statistically significant after washout (−28 ± 4%, *P* < 0.01). Compared with 33 ± 5% in pre-application, the SA was significantly depressed by low-dose Aβ 42 to 22 ± 5% (*P* = 0.01), recovering after washout (27 ± 7%, *P* = 0.530). Compared with 6 ± 1% of the increment in pre-application, the PTP was significantly depressed by low-dose Aβ 42 to −4 ± 1% (*P* = 0.01), again recovering after washout (6 ± 2%, *P* = 0.154). ^*^P < 0.05; ^**^P < 0.01.

In the excitatory neuronal network of the PFC, the F-type connections prominently exhibit forms of short term potentiation (STP) termed SA and PTP (Wang et al., 2006). We next examined effects of low-dose Aβ 40 and Aβ 42 on the SA and PTP in F-connections. Compared with pre-application, the low-dose Aβ 40 to F-connections significantly enhanced synaptic responses during all recording phases, i.e., pre-tetanus baseline, SA induction and PTP induction (Figure 5A1, paired *t*-test with multiple outcome values per connection: all *P* < 0.01, *n* = 4 connected pairs). After washout for 10–30 min, the pre-tetanus EPSPs recovered (*P* = 0.557), however, the enhanced EPSPs still remained at a significantly higher level during the SA and PTP phases (Figure 5A1 inset table, both *P* < 0.01). Thus, on average, the low-dose Aβ 40 enhanced pre-tetanus EPSP by 23 ± 6% (Figure 5A2, *P* = 0.01), which recovered after washout for 10–30 min (*P* = 0.556). Meanwhile, the induction of SA was enhanced nearly 2-fold by the low-dose Aβ 40 (30 ± 13% vs. 16 ± 9% in pre-application, *P* = 0.141) and the enhancement to nearly 4-fold persisted after washout for 10–30 min (58 ± 16% vs. 16 ± 9%, *P* = 0.05). Similarly, the induction of PTP was enhanced more than 2-fold by the low-dose Aβ 40 (5 ± 3% vs. 2 ± 2% in pre-application, *P* = 0.284) and the enhancement to 5-fold persisted after washout (10 ± 2% vs. 2 ± 2%, *P* < 0.01). In the comparison, the borderline absence of statistical significance was likely due to the low sampling of these difficult-to-obtain recordings.

In contrast, low-dose Aβ 42 significantly depressed synaptic responses corresponding to the SA and PTP inductions (Figure 5B1, both *P* < 0.01, *n* = 6 connected pairs). After washout for 10–30 min, the EPSPs comprising the SA and PTP phases of STP were further depressed (both *P* < 0.01). Meanwhile, the EPSPs of the pre-tetanus baseline were also significantly depressed (Figure 5B1 inset table, *P* < 0.01). In Figure 5B2, the opposing actions of low-dose Aβ 42 highly contrast the actions of low-dose Aβ 40 in Figure 5A2. Compared with the pre-tetanus baseline level in pre-application (0 ± 2%), the average EPSP during pre-tetanus recordings became progressively depressed into the recording procedure beginning with the application of low-dose Aβ 42 and into washout (depressed by −6 ± 5%, *P* = 0.08 and by −28 ± 4%, *P* < 0.01, respectively). The average EPSP increment of SA was originally 33 ± 5% in pre- Aβ application. This was significantly reduced to 22 ± 5% following bath application of low-dose Aβ 42 (*P* = 0.05). The average EPSP increment of PTP was 6 ± 1% in pre-application, which became depressed to −4 ± 1% after low-dose Aβ 42 was bath applied (*P* < 0.01). Taken together, effects of low nanomolar concentrations of Aβ 40 and Aβ 42 on EPSP amplitudes are already opposite each other in the pre-tetanus condition, presaging their beneficial and depressing effects on SA and PTP, respectively. These actions on STP induction match their aforementioned influences on isolated EPSP failure rates and on EPSP trains.

## Discussion

The experiments in the current study have explored the effects of soluble monomer predominant extracellular Aβ peptides on synaptic failure rates, synaptic dynamic properties and STP (including SA and PTP) of single excitatory connections in normal PFC. The PFC is highly vulnerable to the effects of aging and neurodegeneration but is relatively understudied in AD. To our knowledge, these are the first whole-cell patch clamp recordings from pairs of individual connections formed by pyramidal neurons in PFC that examine Aβ modulation and toxicity on chemical synaptic transmissions. The advantage of this technique over more conventional field studies is that influences from other afferents and reverberant circuits and influences by exciting neuromodulatory fibers are virtually avoided. In addition, the results are highly repeatable based on single synaptic connections that are classified according to their unitary synaptic dynamics.

We found that the transmission involving individual synaptic connections was significantly enhanced or reduced depending on their intrinsic type (facilitating or depressing), the tested Aβ species (40 or 42 amino acids) and concentration (low dose 1–200 nM vs. high dose 0.3–1 μ M). Our main findings are that bath applications of low nanomolar Aβ 40 have opposite actions on basal and STP properties of F-connections compared with high nanomolar Aβ 40 or low nanomolar Aβ 42. Specifically, when applied to F-connections, low nanomolar Aβ 40 reduces failure rate and enhances EPSP trains and SA and PTP, whereas higher nanomolar Aβ 40 and low nanomolar Aβ 42 alike inhibit them. Interestingly, low nanomolar Aβ 40 inhibits D-connections, acting similarly thereon as high nanomolar Aβ 40 or low nanomolar Aβ 42. In addition, the inhibitory effects of these bath-applied peptides often appeared irreversible despite long-time washout. Nevertheless, reversibility could be demonstrated when Aβ was applied very locally, briefly and followed with a thorough washout.

Normal concentrations of Aβ in CSF and plasma are in the picomolar range (Bohrmann et al., 1999; Teunissen et al., 2002; Lewczuk et al., 2004), but likely higher in the synaptic cleft. Our differential results in the nanomolar range may add new insight into the modulatory role of Aβ 40 by balancing facilitation and depression of synaptic connections to influence activity of synaptic networks in the PFC. Specifically, Aβ 40 at physiological levels moderately reduced EPSP failure rate and significantly enhanced EPSP trains and STP of F-connections while dampening D-connections. The net functional result would be to enhance network activity relevant to working memory while limiting incoming distracting signals, respectively. The first study to show that Aβ 40 actually increased LTP was Wu et al. (1995), moreover the effect was noticed at 200 nM, same as our “low dose” upper limit. In addition, our results add to the notion from other work that Aβ 40 could actually have a beneficial role to moderate Aβ 42 effects (Kim et al., 2007). They are also in line with the differential effects of Aβ 42 and Aβ 25–35 peptides on hippocampal network activation, specifically on θ, β, and γ oscillations (Adaya-Villanueva et al., 2010). However, the possibility is not excluded that Aβ 42 at much lower levels such as in the picomolar range also plays a similar physiological role as does Aβ 40 in synaptic modulation. In a former study of recordings from hippocampal slices, low picomolar concentrations of Aβ 42 caused a marked increase of long-term potentiation in excitatory cells, whereas high nanomolar concentrations lead to the reduction of the potentiation (Puzzo et al., 2008). It may be necessary to study the effects of picomolar Aβ 42 on single synaptic connections in future experiments.

In recent years, it has been found that Aβ is physiologically released from synaptic terminals depending on the levels of synaptic activity (Kamenetz et al., 2003; Cirrito et al., 2005). In turn, Aβ may play an inhibitory feedback role to balance the homeostatsis of neuronal networks (Kamenetz et al., 2003; Hsieh et al., 2006; Venkitaramani et al., 2007). A feature of this feedback loop is that Aβ peptides are eventually cleared by endocytosis and diffusion (Venkitaramani et al., 2007). Our results imply that Aβ 40 at high nanomolar concentrations or Aβ 42 at concentrations as studied here induced an inhibition that might serve as feedback to limit synaptic activity and Aβ production. This is supported by our observation that inhibition of synaptic responses fully recovers when Aβ (40 or 42) is applied briefly and locally followed by a prompt washout (which may be closer to the physiological processes of endocytosis and diffusion). Conversely, inhibition becomes more difficult to recover from after longer-time bath applications of these peptides. These considerations make it likely that the toxic effects of these peptides on synaptic functions become irreversible once they accumulate near synapses to concentrations that overload endocytic and enzymatic removal mechanisms.

The potential toxicity to synaptic function as evidenced by the resistance to recovery following washout could result from the formation of aggregated Aβ oligomers around synapses aided by long bath application times and high concentrations. The Aβ aggregation is dependent on protein concentration and time (Harper and Lansbury, 1997). Aggregated Aβ oligomers may suppress synaptic responses by disrupting synaptic vesicle endocytosis (Kelly and Ferreira, 2007), inhibiting NMDA receptors (Chen et al., 2002) and P/Q-type calcium currents (Nimmrich et al., 2008) and/or via forming artificial ion pores on neuronal membranes (Small et al., 2009). Aβ 40 on the other hand may enhance synaptic facilitation by acting on P-type calcium channels, but once forming oligomers appears to lose the facilitating effect, turning to suppressing synaptic functions (Ramsden et al., 2002). This could explain the opposite effects of Aβ 40 at low vs. high concentrations as observed in the current study. Since Aβ 42 aggregates more readily than the other Aβ species (Snyder et al., 1994), it is not surprising that Aβ 42 might only enhance synaptic activity at picomolar levels (Puzzo et al., 2008). Otherwise, Aβ 42 induces synaptic depression at concentrations at or above the low nanomolar range. In recent years, several studies reported that extrasynaptic NMDA receptors are activated by Aβ oligomers, leading to synaptic dysfunction. Soluble Aβ oligomers increase activation of extrasynaptic NR2B receptors inhibiting NMDAR-dependent LTP (Li et al., 2011). Aβ oligomers also reduce baseline synaptic transmission and spontaneous neuronal network activity and induce retraction of synaptic contacts (Ronicke et al., 2011), some of which may be dependent on extrasynaptic sites of action. Prolonged activation of extrasynaptic NMDAR by Aβ oligomers may also play a key role in pathogenic mechanisms of glutamate excitotoxicity (Stanika et al., 2009), and cell death (Hardingham et al., 2002; Papadia and Hardingham, 2007). While our results pertain to synaptic dysfunction at the resolution of single synaptic connections, future studies are foreseeable to address any extrasynaptic contributions.

An important factor to consider is whether such effects of Aβ peptides on synaptic transmission and plasticity occur at the pre- or post-synaptic element. With our research scheme, this can be speculated upon according to changes in the synaptic dynamic parameters, *D-F-U-A*. The enhancement to F-connections by low-dose Aβ 40 occurs via an increase in the parameter *F*, a presynaptic mechanism. This is further supported by the enhancement to the SA and PTP by the low-dose Aβ 40. It is well-known that synaptic facilitation is mediated by presynaptic residual calcium (Kamiya and Zucker, 1994; Mongillo et al., 2008) and the induction of SA and PTP relies on pre-synaptic mechanisms (Hempel et al., 2000; Zucker and Regehr, 2002). It has also been reported that Aβ acts via presynaptic mechanisms as a positive endogenous modulator for hippocampal synapses in rodent hippocampal cultures and slices (Abramov et al., 2009). The enhancement of F-connections we observe could therefore be related to an effect of Aβ 40 acting on P-type calcium channels, a pre-synaptic calcium channel that mediates synaptic facilitation (Ramsden et al., 2002; Tamse et al., 2003; Iegorova et al., 2010). Both low-dose Aβ 40 and Aβ 42 inhibit D-connections via reducing synaptic strength, reflected in parameter *A*. The *A* parameter represents the synaptic response when the probability of synaptic transmitter release equals 1 at the maximal level. Therefore, changes in synaptic strength, *A*, basically represents alterations in the postsynaptic elements. High-dose Aβ 40 inhibits both F- and D-connections via reducing the release probability, *U*, and the synaptic strength, *A*, which involves both pre- and post-synaptic mechanisms. With respect to the same mechanisms, low-dose Aβ 42 inhibits F-connections. Experimentally, an inhibition on presynaptic transmission is reported after Aβ 42 injection through a block of vesicle fusion in the terminal (Moreno et al., 2009), and the inhibition by Aβ on postsynaptic sites has previously been verified to occur at multiple molecular structures such as AMPA receptors and metabotropic glutamate receptors (Puchtler and Sweat, 1962; Wang et al., 2004; Hsieh et al., 2006; Shemer et al., 2006; Minano-Molina et al., 2011).

Acting on both pre- and post-synaptic sites, Aβ peptides are likely to have multiple actions on multi-synaptic activity in neuronal networks. Low nanomolar concentrations of Aβ 40 significantly enhance synaptic facilitation and both SA and PTP forms of STP of F-connections, meanwhile, inhibiting the synaptic strength (*A*) of D-connections. This finding suggests that Aβ 40 differentially enhances F-connections via presynaptic sites and inhibits D-connections via postsynaptic sites. High nanomolar Aβ 40, on the other hand, inhibits both F- and D-connections through reductions in both *U* and *A* parameters. Aβ 42, even at low nanomolar concentrations, inhibits not only D-connections by reducing *A*, but also to F-connections by reducing both *U* and *A*. These peptides at relatively higher concentrations are therefore expected to play a physiological negative feedback role and/or to produce toxic effects on synaptic functions via both pre- and post-synaptic mechanisms. In addition to pre- and post- synaptic regulation of synaptic activity by physiological levels of Aβ and the depression of excitatory transmission by pathological levels, Aβ peptides are also shown to trigger aberrant synchronous circuit activity, even epileptic discharges, at the network level (Minkeviciene et al., 2009; Palop and Mucke, 2010). Our previous work shows that high levels of soluble Aβ may be involved in aberrant synchronous circuit activity via enhancing neuronal excitability and acting on electrical networks. Here again, physiological levels of Aβ act oppositely playing a negative feedback role to dampen electrical network activity by reducing neuronal excitability (Wang et al., 2009). Future patch clamp recording of inhibitory synaptic connections formed between interneuron and PC pairs can further address this issue from another point of view.

The PFC network has the capacity to support persistent activities during recurrent weak inputs, without resorting to the metabolic expenditure of AP generation, precisely because of some special built-in functions such as synaptic facilitation and predominant STP (Hempel et al., 2000; Wang et al., 2006; Mongillo et al., 2008). Facilitation lasting hundreds of milliseconds (and outlasting depression), SA lasting up to 10 s, and PTP lasting up to minutes are each likely to be important mechanisms to sustain network activity during short-term storage and manipulation processes such as working memory tasks (Magleby, 1987; Fisher et al., 1997; Mongillo et al., 2008). STP is an especially important correlate in the PFC to its integrative functions of working memory as well as in the organization of sequential behavior, mental flexibility and planning (Grafman, 1995; Hempel et al., 2000; Mongillo et al., 2008). Understanding endogenous modulators of the working memory network and its processes is increasingly important to the cognition and neurodegeneration fields. At the resolution of single excitatory synaptic connections, our results show that Aβ may be a homeostatic modulator and play multiple roles depending on intrinsic synapse types, soluble Aβ species and their levels in the synaptic environment. Thus, we predict that Aβ influences persistent neuronal activity during working memory tasks in the PFC. High concentrations and mild accumulation of Aβ around synapses likely lead to declines in memory and cognition such as in the early stages of AD.

### Conflict of interest statement

The authors declare that the research was conducted in the absence of any commercial or financial relationships that could be construed as a potential conflict of interest.

## Appendix

**Table A1:** **Changes of synaptic dynamic properties in the modeling analysis**.

				***A***	***U***	***D***	***F***	***F/D***
Low-doseAβ40	Figure 3A	F-connection	control (*n* = 11)	3.68	0.24	300	589	1.97
			Aβ (*n* = 11)	3.17	0.29	248	926	3.73
			washout (*n* = 9)	3.86	0.16	291	1071	3.69
	Figure 3B	D-connection	control (*n* = 6)	2.61	0.50	359	165	0.46
			Aβ (*n* = 6)	2.21	0.39	623	457	0.73
			washout (*n* = 3)	1.50	0.42	594	382	0.64
High-dose Aβ40&Aβ25–35	Figure 3C	F-connection	control (*n* = 3)	4.68	0.32	235	807	3.43
			Aβ (*n* = 3)	3.92	0.26	243	905	3.73
			washout (*n* = 3)	9.75	0.28	200	896	4.49
	Figure 3D	D-connection	control (*n* = 6)	1.83	0.48	679	149	0.22
			Aβ (*n* = 5)	1.36	0.42	430	465	1.08
			washout (*n* = 5)	1.72	0.34	902	223	0.25
Low-dose Ab42	Figure 3E	F-connection	control (*n* = 7)	5.94	0.39	382	588	1.54
			Aβ (*n* = 7)	5.84	0.26	428	1093	2.55
			washout (*n* = 3)	4.97	0.31	451	964	2.14
	Figure 3F	D-connection	control (*n* = 5)	3.71	0.55	646	119	0.18
			Aβ (*n* = 5) 2.90	0.45	826	114	0.14	
			washout (*n* = 3)	2.54	0.47	825	142	0.17
